# Requirements for and Barriers to Rehabilitation Services for Children With Disabilities in Middle- and High-Income Countries: Scoping Review

**DOI:** 10.2196/50047

**Published:** 2024-08-07

**Authors:** Yijun Xie, Jing Wu, Yao Li, Hui Liu, Yanyan Peng, Ping Zhou, Yizhou Sun, Luyan Kang, Chenghua Jiang, Hengjing Wu

**Affiliations:** 1 Clinical Center for Intelligent Rehabilitation Research Shanghai Yangzhi Rehabilitation Hospital (Shanghai Sunshine Rehabilitation Center), School of Medicine Tongji University Shanghai China; 2 Department of Fundamental Nursing School of Nursing Shanghai University of Traditional Chinese Medicine Shanghai China; 3 Office of Rehabilitation Shanghai Disabled Persons' Federation Shanghai China

**Keywords:** children with disabilities, barriers, health services, middle- and high-income countries, child, low income, middle income, disability, children, disabilities, income, barrier, rehabilitation, suitability, availability, affordability, support system, support, awareness, policy

## Abstract

**Background:**

The rehabilitation of children with disabilities has received considerable attention from the United Nations. However, the state of rehabilitation services for children with disabilities worldwide remains far from optimistic, even in economically affluent middle- and high-income countries.

**Objective:**

This scoping review aimed to identify the rehabilitation needs of children with disabilities and their barriers to rehabilitation services in middle- and high-income countries.

**Methods:**

A systematic search was conducted using MEDLINE and Web of Science for papers published from January 2013 to December 2023. Studies were included if they were peer-reviewed, full-text articles related to children with disabilities, reporting on their access to rehabilitation services, and conducted in countries classified by the World Bank 2023 as middle- and high-income economies. Exclusion criteria included duplicates, unavailable full texts, and studies without distinct outcomes. A total of 27 studies were selected following PRISMA (Preferred Reporting Items for Systematic Reviews and Meta-Analyses) guidelines, focusing on children, their families, or service providers.

**Results:**

The suitability, availability, and affordability of rehabilitation services were identified as the major needs and barriers for children with disabilities in middle- and high-income countries. This included communication barriers, a need for more personnel and facilities, and the stagnation and inadequacy of economic subsidies.

**Conclusions:**

Middle- and high-income countries have relatively well-established rehabilitation infrastructure and support systems. They are nevertheless insufficient for meeting the needs of children with disabilities. More attention should be paid to these issues to improve the well-being of children with disabilities. The data provided by this review can help raise awareness of rehabilitation needs and barriers at the policy level.

## Introduction

Worldwide, over 240 million children are impacted by disabilities [1], which predominantly manifest as functional impairments. These impairments substantially inhibit their ability to engage in foundational activities such as learning, daily functions, and social integration. Defined by age, individuals younger than 18 years who experience physiological, psychological, or cognitive deficits, whether congenital or acquired, are classified as children with disabilities [2]. Children are in a crucial period of growth and development. Timely intervention and treatment not only promote the physical and cognitive development of children with disabilities but also minimize the impact of disabilities on their lives to the greatest extent possible [3]. Rehabilitation for children with disabilities encompasses a comprehensive approach that integrates medical, educational, and social rehabilitation methods. Its primary aim is to assist children in overcoming physiological, psychological, or societal barriers. The ultimate goal is to maximize their quality of life, functionality, and autonomy [4]. Compared with their healthy peers, children with disabilities have higher rates of morbidity, require increased medical attention, and have longer hospital stays [5]. The rehabilitation needs of children with disabilities are more pressing. However, children with disabilities often face greater challenges in meeting their rehabilitation needs. Children with disabilities sometimes face the impact of different social attitudes and cultural traditions, experiencing discrimination and exclusion, which results in their service needs being overlooked [5]. Furthermore, barriers related to transportation and the natural environment also limit the fulfillment of the needs [6].

Rehabilitation welfare policies for children with disabilities have been established in most regions [7-9]. However, these policies seem to have not yielded satisfactory outcomes [10]. Children with disabilities still face considerable drawbacks in various indicators used to assess child welfare. In sub-Saharan African countries [11], common deficiencies, such as attitudinal problems, poverty, inadequately trained, health care professionals, and physical inaccessibility are seen. The existence of regional disparities contributes to the insufficient distribution of health care resources and funding [12,13]. This further affects the accessibility of rehabilitation services for children with disabilities residing in different regions. The disparity in national economic levels directly impacts the allocation of medical resources and funding, resulting in significant variations in the level of support available for children with disabilities residing in different regions [14,15]. The World Bank classifies the global economy into 4 distinct categories based on 3 income thresholds: US $1135, US $4465, and US $13,846 per capita gross national income (GNI) for the year 2022. These categories are low-income, lower-middle-income, upper-middle-income, and high-income [16]. Previous research has predominantly focused on countries classified as low-income economies [12,17,18]. Even in middle- and high-income countries with relatively better economic conditions, the status of rehabilitation support for children with disabilities is equally concerning [19]. Compared with low-income countries, these nations and regions possess a greater abundance of resources and experience in providing rehabilitation services. However, there are still issues with the accessibility of rehabilitation services for children with disabilities in these regions [17]. The growth of the population and the increasing prevalence of chronic illnesses have raised additional demands for rehabilitation services. Disparities in economic levels have exacerbated regional inequalities in the allocation of rehabilitation resources [18]. Additionally, differences between early-established welfare policies and current rehabilitation needs also impact the fulfillment of these demands [20]. There has been relatively limited exploration in existing literature regarding the specific influencing factors that pertain to middle- to high-income countries and regions. The purpose of this study was to systematically review the existing literature on the state of rehabilitation services for children with disabilities, especially in middle- and high-income countries. This review aims to explore the factors influencing the accessibility and effectiveness of these services and to identify gaps in current policies that could be addressed to better meet the diverse needs of children with disabilities across different economic contexts.

## Methods

### Review Design

We conducted a scoping review to map the evidence regarding the accessibility of rehabilitation services for children with disabilities across the world. The review was based on a systematic search of relevant sources that have been used previously in the field of disability [21,22]. Scoping reviews have broad, comprehensive objectives compared with systematic reviews, which are often guided by more narrow, focused research questions [23]. We chose this approach given the limited range of rigorous study designs described in traditional systematic review papers. In addition, because this scoping review was intended to provide a descriptive overview of the applicable literature [24], we did not critically assess the papers. We acknowledge that the studies included in the review may have methodological strengths and limitations. Therefore, we approach the selection and analysis with caution. We are particularly mindful that some studies may not be methodologically suited to provide precise explanations. However, we make an effort to highlight the contributions of each study and their role in the broader context. While scoping reviews may not be the most robust tool for evidence synthesis, they provide us with a valuable starting point to understand the unmet health care needs of children with disabilities and point the way for future research. The outline for this review followed the 5-stage framework described by Arksey and O’Malley [23], which includes (1) identifying the research question, (2) identifying relevant studies, (3) selecting studies, (4) charting the data, and (5) accumulating, summarizing, and reporting the results.

### Identifying the Research Question

The purpose of this scoping review was to understand the current status of rehabilitation services for children with disabilities, explore the influencing factors, and make constructive observations. This is important for improving the well-being of children with disabilities and responding to the World Health Organization’s (WHO’s) 2030 Rehabilitation Initiative.

### Identifying Relevant Studies

We searched the electronic databases MEDLINE and Web of Science using combinations of the keywords, which are Rehabilitation, Disabled Children, and Health Services for Persons with Disabilities. Specific search strategies can be found in Multimedia Appendix 1. We screened the titles and abstracts of publications from the past 10 years (2013-2023) to find relevant articles and capture the most recent evidence. The literature within this time frame encompasses alterations in policy, technology, and rehabilitation services, thereby augmenting the filterability and analyzability of research data and literature [25-27]. Through this strategy, we aimed to provide highly relevant and practical information.

### Eligibility Criteria and Study Selection

Studies were eligible if they met the following criteria: (1) a study related to a child with a disability; (2) the participants were children with disabilities, their family members (parents, relatives), or staff providing services; (3) the study reported the disabled child’s access to rehabilitation services; and (4) the study was conducted in countries classified by the World Bank 2023 as middle- and high-income economies. Studies were excluded if the full text of the electronic source was not available. Duplicate reports of the same study were combined if they reported different results or excluded if they had the same results. The first round of screening removed duplicate studies and eliminated articles based on titles and abstracts. Our selection process was conducted in 2 stages to ensure accuracy and relevance. In the first stage, XYJ and LY independently reviewed titles, abstracts, and full texts of identified publications. Each conducted their assessments separately to maintain objectivity, documenting their findings to ensure alignment with our inclusion criteria.

In the second stage, these authors met to jointly reassess the retained papers, focusing specifically on their relevance to our review’s topic. This collaborative review helped refine the selection further. Any disagreements encountered during this reassessment were resolved through detailed discussions until a consensus was reached regarding each paper’s compliance with the inclusion criteria.

### Data Charting

Participant demographics (eg, information on children with disabilities and type of disability), access to rehabilitation services, and study-related data (eg, the author or researcher, year of publication, study objectives, and whether the study was in a middle- or high-income country) were extracted and put into Excel (Microsoft Corp). Two independent reviewers conducted the extraction of pertinent data from the papers included in the scope-defining review. Next, we read the full texts of the remaining papers and extracted the following: the author or researcher, date of publication, study objectives, inclusion criteria, age of child or children, sample size, age of parents, type of disability, city or country or setting of the study, research method, study results, and the facilitators of and barriers to rehabilitation services. Any disagreements among reviewers that cannot be resolved through discussion or consensus will be resolved by a third reviewer. Penchansky and Thomas [28] have compiled a set of specific dimensions that delineate the relationship between patients and the health care system. Their analysis demonstrates discernible distinctions among these dimensions. These specific dimensions encompass the aspects of availability, accessibility, accommodation, affordability, and acceptability. The extracted information has been categorized using these 5 dimensions.

## Results

### Search Results

**Figure 1:**
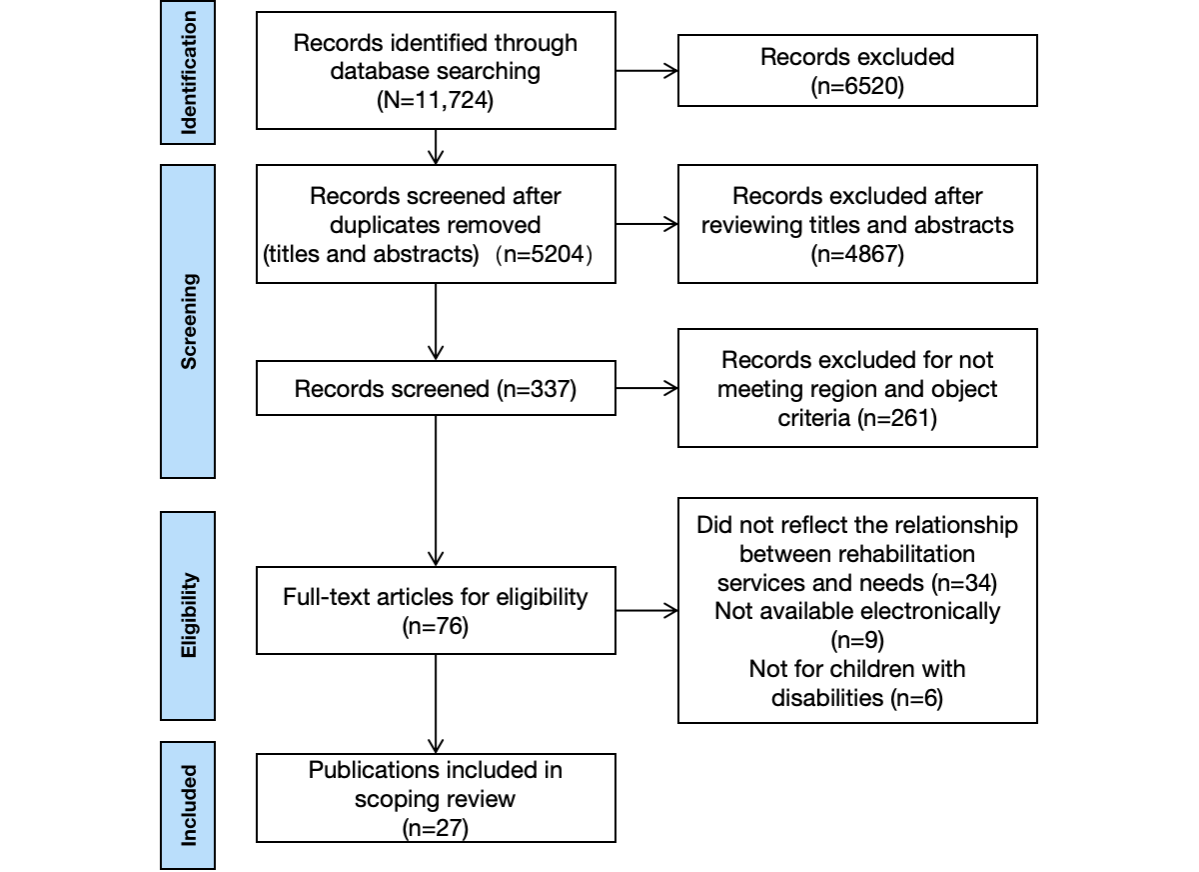
PRISMA (Preferred Reporting Items for Systematic reviews and Meta-Analyses) flow diagram of the study selection process.

The initial search produced 11,724 documents after removing 6520 duplicates; 4867 documents were further removed after screening titles and abstracts. We then screened 76 full-text papers and excluded an additional 49 papers. The reasons for exclusion during full-text screening were the following: (1) did not reflect the relationship between rehabilitation services and need (n=34); (2) full text was not available electronically (n=9); and (3) did not target children with disabilities (n=6). Figure 1 illustrates the search process. All the included studies are listed in Multimedia Appendix 2 [15,29-54].

### Characteristics of the Included Articles

The majority of the studies (21/27, 77.8%) of children’s rehabilitation services were conducted in high-income economies, with 22.2% (6/27) at upper-middle income levels. Regarding regions, the highest number of studies were conducted in East Asia and Pacific (9/27, 33.4%) and Europe and Central Asia (8/27, 29.6%), followed by North America (7/27, 25.9%), Middle East and North Africa (2/27, 7.4%), and sub-Saharan Africa (1/27, 3.7%).

The selected studies investigated the rehabilitation needs of children with disabilities and the barriers they face. Of these studies, 33.4% (9/27) solely involved children with disabilities, while the remaining papers gathered information from parents of children with disabilities (12/27, 44.4%) or professional caregivers (4/27, 14.8%). In some studies, children with disabilities could not articulate their needs due to their unique physical conditions. Consequently, researchers gained insights into the children’s rehabilitation requirements and challenges by conducting interviews with their parents. For instance, parents were asked about their perceptions of their child’s experience with rehabilitation services or to elaborate on the effect of their child’s disability on the family’s quality of life.

Overall, the included studies had diverse goals, such as investigating the unmet health care needs of children with disabilities, insurance plans, quality of life for children and families, participation in activities, and intervention programs. Nevertheless, the primary focus of the reviewed papers was on the health care requirements of children with disabilities and the barriers they face, with relatively limited attention given to treatment techniques or clinical outcomes. Table 1 lists the descriptive statistics of the included documents. All selected documents will be included as supplementary materials in the form of appendices.

**Table 1:** General characteristics of the studies eligible for inclusion in the review.

Characteristics		Publications
**World Bank income category, n (%)**		
	High-income economy	21 (77.8)
	Upper-middle income economy	6 (22.2)
**World Bank region, n (%)**		
	East Asia and Pacific	9 (33.4)
	Europe and Central Asia	8 (29.6)
	North America	7 (25.9)
	Middle East and North Africa	2 (7.4)
	Sub-Saharan Africa	1 (3.7)
**Participants, n (%)**		
	Parent	12 (44.4)
	Children	9 (33.4)
	Caregiver	4 (14.8)
	Multiple	2 (7.4)
**Disability domain, n (%)**		
	Multiple domains	17 (62.9)
	Physical	5 (18.5)
	Intellectual	2 (7.4)
	Neurological	2 (7.4)
	Hearing	1 (3.7)
**Study design, n (%)**		
	Quantitative	16 (59.3)
	Qualitative	10 (37)
	Mixed methods	1 (3.7)

### Rehabilitation Needs and Barriers to Accessing Services

The scale by Penchansky and Thomas [28] addresses access to health care and consists of 5 categories, which include accessibility, acceptability, affordability, appropriateness, and availability. This classification’s validity has been confirmed through interview data analysis. Accessibility describes the physical connection between the family and the location of rehabilitation services. Acceptability refers to clients’ and providers’ satisfaction with health care services. Affordability relates to the connections between financial constraints, financial capacity, and health insurance availability. Appropriateness refers to the match between supply and patient demand. Availability describes the connection between supply and demand for resources. This study used Penchansky’s scale to identify information about barriers to rehabilitation services (Table 2).

**Table 2:** Factors affecting the fulfillment of rehabilitation needs of children with disabilities.

Factors		Reference
**Accessibility**		
	Transportation problems	Jeong [40]
	Distance to service	Alyami et al [29], Mulligan et al [41], Caicedo [36]
**Acceptability**		
	Rejection and discrimination	Wang et al [39]
	Privacy and trust	Teleman et al [42]
	Shame and fear	Mulligan et al [41], Teleman et al [42], Wang et al [39]
	Doubts about validity	Teleman et al [42], Sukeri et al [37], Lindly et al [43], Xia et al [44]
**Affordability**		
	Insurance issues	Lindly et al [43], Schaible et al [30], Robinson et al [33], Xia et al [44]
	Burden of treatment	Alyami et al [29], Lindly et al [43], Parr et al [32], Roux-Levy et al [38], Piškur et al [45], Xia et al [44], Houtrow et al [46], Raouafi et al [15]
	Other economic issues	Lindly et al [43], Schaible et al [30], Robinson et al [33], Gallagher et al [34], Raouafi et al [15], Jeong [40], Meehan et al [47], Umat et al [48]
**Appropriateness**		
	Number of treatments	Alyami et al [29], He et al [49]
	Targeted rehabilitation services	Sukeri et al [37], Wang et al [39]
	Expertise	Mulligan et al [41], Sukeri et al [37], Roux-Levy et al [38], Wang et al [39]
	Communication barriers	Carter et al [50], Mulligan et al [41], Sukeri et al [37], Parr et al [32], Caicedo [36], Ziviani et al [35], Pérez-Ardanaz et al [51]
**Availability**		
	Procedures	Roux-Levy et al [38], Sukeri et al [37]
	Schedule	Wang et al [39], Alyami et al [29], Ziviani et al [35]
	Messaging	Xia et al [44], Alyami et al [29], Matsuzawa and Shiroki [52], Ziviani et al [35], Sukeri et al [37]
	Spiritual support	Xia et al [44], Alyami et al [29], Matsuzawa and Shiroki [52], Mulligan et al [41], Piškur et al [45], Cacioppo et al [31], Lindly et al [43], Khusaifan and Keshky [53], Umat et al [48]
	Equipment service supply	Carter et al [50], Pérez-Ardanaz et al [51], Alyami et al [29], Mulligan et al [41], Houtrow et al [46], Roux-Levy et al [38], Arabiat et al [54], Jeong [40], Sukeri et al [37]

#### Accessibility

The accessibility of rehabilitation services refers to the reduction of various barriers that may exist in accessing these services, intending to achieve equality for all. Ensuring that children with disabilities can easily access rehabilitation services has a significant impact on meeting the rehabilitation needs of these children [55]. On the one hand, geographic distance is strongly linked with time costs for children with disabilities to access rehabilitation [29]. On the other hand, the distance barrier also interferes with the ability of rehabilitation specialists to connect and share experiences. Geographical isolation hinders information exchange in resource-poor regions, often leaving rehabilitation experts in these areas isolated and unsupported “information islands” [27]. Apart from the challenges associated with the coverage and frequency of “public transportation,” Jeong [40] found that “personal transportation” includes factors such as the availability of vehicles also influencing the accessibility of rehabilitation services.

#### Acceptability

The acceptance of rehabilitation services by children with disabilities is affected by personal factors such as stigmatization and doubts about service effectiveness [28,29,55,56]. According to 3 papers [39,41,42], disability is often stigmatized, leading to a reluctance among parents to seek rehabilitation treatment for their children with disabilities. This stigma associated with disability can deter families from pursuing the necessary care and therapies [57]. Discrimination may also lead to rehabilitation professionals refusing to provide their services. This directly impacts the trust relationship between children with disabilities and service providers, ultimately influencing the decision to seek rehabilitation services [30]. In a reviewed study [58], a Chinese mother faced discrimination due to her child’s autism, leading to trust issues in rehabilitation services and service discontinuation. The current situation of this discrimination may explain how children with disabilities are deprived of their rehabilitation rights through voluntary abandonment in a so-called equitable institutional environment. Furthermore, rehabilitation care is a long-term process. Short-term outcomes are often limited. Some families of children with disabilities may develop a negative attitude toward rehabilitation services because they perceive that the short-term impact falls short of their initial expectations [56].

#### Affordability

The relationship between disability and poverty is reciprocal [56,57]. Families of children with disabilities were more likely to experience financial burdens owing to their child’s health condition [30]. Such families face multiple financial challenges, including rehabilitation expenses [30,58,59], assistive equipment, institutional care [29], home renovations [15], and travel expenses [60]. While governments in middle- to high-income countries generally offer financial assistance and insurance, some families having children with disabilities still experience significant economic burdens [61-63], and current insurance benefits fall short of completely covering the rehabilitation needs of children with disabilities [31]. The gap between financial assistance and the actual needs continues to widen [32,33]. It is worth noting that the current financial assistance system primarily focuses on children with confirmed disabilities. This might exclude children with disabilities who require rehabilitation but do not have a formal diagnosis [34].

#### Appropriateness

Effective rehabilitation treatment relies heavily on the appropriateness of rehabilitation services, which includes matching treatment services, goals, and assistive devices with patients’ needs [64]. As reported by mothers in a study conducted in Kelantan, Malaysia, profit-oriented organizations may overlook the individual needs of children with disabilities, which can diminish the effectiveness of rehabilitation services. Parents have noted that insufficient responsiveness from professionals has reduced the practical utility of the services [56]. The professionalism of rehabilitation professionals is crucial for the appropriateness of services. Insufficient professionalism can result in delays in addressing the conditions of children with disabilities. Rehabilitation training is widely available in middle- to high-income countries. However, there is a lack of professional ability among rehabilitation personnel. This deficiency leads to children with disabilities not receiving timely and accurate treatment, even when they diligently follow their rehabilitation plans [56]. Due to limitations in the size and staff of rehabilitation institutions, the frequency and number of treatments can also impact the effectiveness and satisfaction of rehabilitation services. In a family survey of Saudi Arabian children with hearing impairment [26], more than half of the participants believed that the number of treatment sessions provided was inadequate to meet their rehabilitation demands.

Communication gaps between the staff of rehabilitation institutions and the parents of children with disabilities can lead to misunderstandings and negative perceptions [35,36]. A lack of collaborative communication among staff within rehabilitation institutions can increase feelings of isolation among rehabilitation professionals. It also has the potential to reduce job motivation [28]. Even though rehabilitation facilities in middle- and high-income countries are relatively well-established, the lack of communication and interaction has still been identified as a serious issue. Studies indicate that poor internal and external communication within institutions, along with a lack of collaboration, increases the co-ordination burden on parents of children with disabilities [58]. This also hinders the transfer of treatment between different rehabilitation systems and the sustainable development of children with disabilities [32]. The compatibility of assistive facilities is also a key factor. For instance, in the provision of rehabilitation equipment for children with physical disabilities, nonportable wheelchairs are often voluntarily abandoned because they do not meet the actual needs of children with disabilities [37].

#### Availability

The availability of rehabilitation services for children with disabilities emphasizes the presence and provision of rehabilitation resources, as well as the reasonable scheduling of rehabilitation service delivery, which are key factors in meeting the rehabilitation needs of children with disabilities. Factors related to this include cumbersome procedures, the need for information and emotional support, the need for specialized medical facilities, and scheduling convenience. If the family cannot afford the cost of the wait times, they will give up on rehabilitation [38]. Australian parents of children with physical disabilities report that flexible scheduling and location of rehabilitation services provide them with convenience [35].

Studies highlight the need for information on rehabilitative options [29]. Available research showed that middle- and high-income countries have relatively well-established rehabilitation facility structures, yet the exchange and communication of rehabilitation information have not received adequate attention. The quantity and quality of information provided by rehabilitation service institutions are often unsatisfactory. This compels parents of children with disabilities to seek solutions on their own [58]. It is worth noting that children with disabilities and their caregivers experience both physical and mental stress. While it has been proven that psychological support is beneficial for their mental well-being [26,40,41], it remains one of the common unmet needs within rehabilitation programs [42,57]. Regional disparities in rehabilitation services create challenges for children with disabilities who seek higher-quality rehabilitation services [38]. To access higher-level services, they are compelled to bear higher treatment costs [29,39,65]. The current situation of oversaturated health care resources and low-quality health system services also prolongs the access cycle and reduces the level of services for children with disabilities [38,55].

The most commonly discussed issue in the literature was availability, followed by affordability. Notably, Europe or Central Asia, North America, and East Asia or Pacific were the main regions reporting both of these issues.

High-income countries reported the greatest need for specialized facilities and emotional support, followed by financial issues not covered by insurance and treatment costs. Communication barriers and doubts about the effectiveness of rehabilitation treatment were also highlighted as acceptability concerns. Professionalism and access to spiritual support were also mentioned. In contrast, participants from middle-income countries expressed greater concern with the accessibility, appropriateness, and acceptability of rehabilitation services. This disparity might be related to the fact that half of the studies in middle-income countries focused on rehabilitation service workers.

## Discussion

### Principal Findings

This scoping review aimed to provide an overview of the rehabilitation needs and related obstacles faced by children with disabilities residing in middle- and high-income countries. The final review comprised 27 peer-reviewed papers published between 2013 and 2023, categorized into 5 groups based on rehabilitation-related factors, allowing for a summary and comparison of related studies. The findings of this review suggest that even in economically prosperous middle- and high-income countries, common factors still affect children with disabilities’ achievement of rehabilitation services. The appropriateness, accessibility, and affordability of rehabilitation services were identified as the main factors related to the needs and barriers of families of children with disabilities. Having identified these factors, it is possible to propose recommendations aimed at the formulation of intervention strategies.

The accessibility of rehabilitation services is a fundamental issue related to the presence or absence of service resources. In line with research conducted in low-income countries, it is consistent that children with disabilities generally face difficulties in accessing adequate health care services [66]. This issue is not only confined to low-income countries but also in middle- and high-income countries with more developed health care systems [67]. However, there are differences between the 2 when it comes to the specific challenges they face: unlike the generally low availability of rehabilitation resources in low-income countries, the main influence factor in middle- and high-income countries is the uneven distribution. The availability of rehabilitation resources is generally high but concentrated in economically developed areas [68]. In underdeveloped regions, children with disabilities face challenges in accessing rehabilitation resources, resulting in a relative scarcity of rehabilitation resource provision [28]. Capitalizing on the spillover effect of developed regions, rehabilitation resources should be strategically allocated to less developed regions. This can improve the overall quality of services for families of children with disabilities, enhancing the capabilities of existing health care and rehabilitation professionals while attracting new talent to underserved areas. By increasing both the quantity and quality of professionals, the accessibility and effectiveness of services could be improved. Through the implementation of these measures, we can mitigate the uneven distribution of resources between regions and reduce disparities in rehabilitation levels [68].

Access to affordable and adequate health care for children with disabilities remains a pervasive challenge in middle- and high-income regions. It was discussed in 12 papers in 8 different countries. In contrast to low-income countries, the economic pressure in this context does not arise from a lack of assistance but rather from the effectiveness of the assistance system itself [26,35,64]. As economic levels and rehabilitation costs continue to rise, the actual medical expenses surpass the financial support available to families. This discrepancy has rendered the previously established financial subsidy policies inadequate in meeting the current rehabilitation support needs. This diminishes the effectiveness of assistance. Although such countries generally have relatively completed insurance systems and benefit arrangements [25], an unmet need for support also exists. For instance, current insurance benefits fall short of completely covering rehabilitation costs for children with disabilities [39]. Evaluating the effectiveness of financial assistance programs and adjusting subsidy standards for children with disabilities based on actual circumstances is necessary. By understanding the specific needs of different types of children with disabilities and making targeted adjustments to subsidy policies, resource allocation can be improved, leading to enhanced utilization efficiency. Extending insurance coverage to encompass a diverse range of rehabilitation-related expenditures will better meet the individualized needs of children with disabilities. Besides traditional physical and speech therapy, occupational therapy, psychological counseling, and special education resources should also be incorporated. Achieving this goal necessitates close co-operation among health care service providers, policymakers, and other stakeholders to adopt policies to address complex rehabilitation needs. Simultaneously, the long-term and intricate nature of the rehabilitation process for children with disabilities must be considered, and adjustments in assistance to offer more flexible and enduring support should be made.

Effective communication is a pivotal issue in improving the appropriateness of rehabilitation for children with disabilities. In low-income countries, this issue has received relatively little attention in research [12]. However, in this review, 6 out of 15 middle- to high-income countries (40%) have reported this concern. This encompasses the need for communication between children with disabilities and rehabilitation staff, knowledge exchange among rehabilitation professionals, and referrals between rehabilitation institutions. Educating and training rehabilitation service providers in effective communication strategies is crucial, encouraging them to actively listen to families’ opinions and address their concerns. Future research should delve into the collaboration between rehabilitation experts and institutions, emphasizing resource sharing, information flow, and referral and treatment models for children with disabilities. This approach aims to enhance the sustainability of rehabilitation services for children with disabilities.

This review has several limitations that should be noted. First, even though we used an extensive search strategy to find all relevant studies, the search scope was constrained because our study was not a systematic review. Second, most studies were conducted in North America, Europe, and Asia or the Pacific, making comparisons by continent and economic development level difficult. Furthermore, some of the studies were carried out at the district level, making it difficult to extrapolate to the nation’s overall rehabilitation status. Finally, rather than focusing on more general disability identification, interventions, and clinical research, we mainly considered the rehabilitation needs of children with disabilities and the related barriers. Therefore, this might not reflect access to rehabilitation services in the broadest sense, as mentioned in WHO’s 2030 Rehabilitation Initiative. Future research could further refine rehabilitation initiatives related to children with disabilities to obtain more complete information.

### Conclusion

This scoping review highlighted the rehabilitation needs of children with disabilities and the barriers to rehabilitation services in middle- and high-income countries. The affordability, availability, and appropriateness of rehabilitation services were major factors affecting access. Despite the established service supply in these countries, access falls short of meeting the needs of children with disabilities. To address these challenges, it is necessary to expand financial coverage, improve infrastructure and professional training, and strengthen supporting institutions.

## Appendix

Multimedia Appendix 1 Retrieval strategy.

Multimedia Appendix 2 Included literature.

Multimedia Appendix 3 PRISMA-ScR (Preferred Reporting Items for Systematic Reviews and Meta-Analyses extension for Scoping Reviews) checklist.

## Abbreviations

GNI: gross national income
PRISMA: Preferred Reporting Items for Systematic Reviews and Meta-Analyses
WHO: World Health Organization

## Data Availability

All data generated and analyzed during this study are included in this published paper and its Multimedia Appendices.
