# Supplementary material for: Requirements for and Barriers to Rehabilitation Services for Children With Disabilities in Middle- and High-Income Countries: Scoping Review
Source: Interact J Med Res. 2024 Aug 7;13:e50047. doi: 10.2196/50047 (PMC11339577; doi:10.2196/50047)
Supplement: Multimedia Appendix 1 [file ijmr_v13i1e50047_app1.pdf]

Multimedia Appendix 1: Retrieval Strategy

Medline	Web of Science
(((((((((Children with Disabilities) OR (Children with Disability)) OR (Disability, Children with)) OR (Children, Disabled)) OR (Handicapped Children)) OR (Children, Handicapped)) OR (Child, Disabled)) OR (Disabled Child)) OR ("Disabled Children"[Mesh])) AND ((((Health Services for the Disabled) OR (Health Services for People with Disabilities)) OR (Health Services for Disabled Persons)) OR (Habilitation))) AND (("Rehabilitation"[Mesh]) OR (Habilitation))	(((((((((((TS=(Children with Disabilities) OR TS=(Children with Disability)) OR TS=(Disability, Children with)) OR TS=(Children, Disabled)) OR TS=(Handicapped Children)) OR TS=(Children, Handicapped)) OR TS=(Child, Disabled)) OR TS=(Disabled Child)) OR TS=(Health Services for the Disabled)) OR TS=(Health Services for People with Disabilities)) OR TS=(Health Services for Disabled Persons)) OR TS=(Habilitation)) AND TS=(Rehabilitation)) OR TS=(Habilitation)
