# Supplementary material for: Requirements for and Barriers to Rehabilitation Services for Children With Disabilities in Middle- and High-Income Countries: Scoping Review
Source: Interact J Med Res. 2024 Aug 7;13:e50047. doi: 10.2196/50047 (PMC11339577; doi:10.2196/50047)
Supplement: Multimedia Appendix 2 [file ijmr_v13i1e50047_app2.pdf]

Multimedia Appendix 2: Included Literature

Year	Study	Country	Region	Country Type	Disability Type	Objects	Research type
2022	Wang et al. [50]	China	East Asia and Pacific	MICs	Mutiple	Caregiver	Qualitative
2022	Matsuzawa et al.[63]	Japanese	East Asia and Pacific	HICs	Mutiple	Parent	Qualitative
2021	Carter et al.[61]	Britain	Europe and Central Asia	HICs	Physical	Children	Qualitative
2021	Cacioppo et al.[40]	France	Europe and Central Asia	HICs	Mutiple	Parent	Quantitative
2021	Roux-Levy et al. [48]	France	Europe and Central Asia	HICs	Intellectual	Parent	Mixed methods
2021	Khusaifan et al.[64]	Saudi Arabia	Middle East and North Africa	HICs	Mutiple	Parent	Quantitative
2021	Teleman et al. [53]	Sweden	Europe and Central Asia	HICs	Mutiple	Multiple	Qualitative
2020	Xia et al.[55]	China	East Asia and Pacific	MICs	Mutiple	Caregiver	Quantitative
2020	Gallagher et al.[43]	Ireland	Europe and Central Asia	HICs	Mutiple	Children	Quantitative
2020	Jeong et al.[51]	Korea	East Asia and Pacific	HICs	Mutiple	Children	Qualitative
2020	Pérez-Ardanaz et al.[62]	Spain	Europe and Central Asia	HICs	Physical	Children	Quantitative
2020	Robinson et al.[42]	United States of America	North America	HICs	Mutiple	Children	Qualitative
2020	Houtrow et al.[57]	United States of America	North America	HICs	Mutiple	Parent	Quantitative
2019	Umat et al.[59]	Malaysia	East Asia and Pacific	MICs	Mutiple	Parent	Quantitative
2018	Arabiat et al.[65]	Australia	East Asia and Pacific	HICs	Physical	Parent	Quantitative
2018	Raouafi et al.[15]	Canada	North America	HICs	Neurodisability	Children	Quantitative
2018	Schaible et al.[33]	United States of America	North America	HICs	Physical	Parent	Quantitative
2017	He et al.[60]	China	East Asia and Pacific	MICs	Intellectual	Children	Quantitative
2017	Sukeri et al.[47]	Malaysia	East Asia and Pacific	MICs	Mutiple	Parent	Qualitative
2017	Mulligan et al.[52]	South Africa	Sub-Saharan Africa	MICs	Mutiple	Caregiver	Qualitative
2016	Alyami et al. [30]	Saudi Arabia	Middle East and North Africa	HICs	Hearing	Children	Quantitative
2016	Lindly et al.[54]	United States of America	North America	HICs	Mutiple	Children	Quantitative
2016	Meehan et al.[58]	United States of America	North America	HICs	Physical	Multiple	Quantitative
2015	Piškur et al.[56]	Netherlands	Europe and Central Asia	HICs	Mutiple	Parent	Quantitative
2014	Ziviani et al.[45]	Australia	East Asia and Pacific	HICs	Mutiple	Parent	Qualitative
2014	Caicedo et al.[46]	United States of America	North America	HICs	Mutiple	Parent	Qualitative
2013	Parr et al.[41]	Britain	Europe and Central Asia	HICs	Neurodisability	Caregiver	Quantitative
